# Flow cytometric analysis of CD64 expression pattern and density in the diagnosis of acute promyelocytic leukemia: a multi-center study in Shanghai, China

**DOI:** 10.18632/oncotarget.20814

**Published:** 2017-09-11

**Authors:** Min Liu, Xiangqin Weng, Shenglan Gong, Hui Chen, Jing Ding, Mengqiao Guo, Xiaoxia Hu, Jianmin Wang, Jianmin Yang, Gusheng Tang

**Affiliations:** ^1^ Department of Hematology, Changhai Hospital, The Second Military Medical University, Shanghai, China; ^2^ Institute of Hematology, Ruijin Hospital, Jiaotong University, Shanghai, China

**Keywords:** acute promyelocytic leukemia (APL), APL diagnostic immunophenotypic panel, flow cytometry, diagnostic performance

## Abstract

No unified immunophenotypic profiles and corresponding analytic strategies have been established for the rapid diagnosis of acute promyelocytic leukemia (APL) using flow cytometry (FCM). Here we describe a characteristic immunophenotypic panel that can rapidly and accurately distinguish APL from other types of adult acute myeloid leukemia (AML) using only FCM. By comparing APL cells and non-APL AML cells that share APL common immunophenotypes (CD34^−^CD117^+^HLA^−^DR^−^) we found that CD64 was a significant factor that differentiated APL from other AMLs. Further retrospective analyses of 205 APL and 629 non-APL AML patients from different hematology centers showed that either the CD64^dim and homo^CD13^+homo^ CD33^+homo^MPO^+^ (myeloperoxidase) CD11c^−^ panel or the CD64^dim and homo^CD13^+homo^ CD33^+homo^MPO^+^ CD11c^+^CD10^−^CD117^+^ SSC^high^ (high side scatter signal) panel could distinguish APL from non-APL AML patients with nearly 100% sensitivity, specificity and accuracy. Moreover, relative quantification of CD64 expression enhanced the applicability of our APL diagnostic immunophenotypic panels (ADI-panels) in different hematology centers. Application of the ADI-panels will decrease diagnosis time and improve personalized treatment for APL, a life-threatening disease with very rapid progression.

## INTRODUCTION

Acute promyelocytic leukemia (APL) is a highly aggressive disease that accounts for 6−8% of all adult acute myeloid leukemia (AML) [1]. Childhood APL accounts for approximately 10% of AML in the United States and nearly 30% in China [2]. Without prompt early diagnosis and highly effective intervention with all-trans retinoic acid, APL typically develops with an accompanying risk of life-threatening coagulopathy. Leukemia diagnosis relies on combinatorial analyses of morphology, immunology, cytology, and molecular biology (MICM). However, a definitive morphologic diagnosis is difficult in clinical practice, especially because of morphologic variants and inadequate aspirate smears. Furthermore, fluorescence *in situ* hybridization (FISH) and reverse transcriptase-polymerase chain reaction (RT-PCR) analyses for the detection of abnormal RARα fusion genes (e.g. PML-RARα, NPM-RARα) are typically performed only on suspicious cases [3, 4]. Moreover, cytogenetic analysis of the t(15;17)(q22;q21) and other rare variant chromosome translocations using karyotyping is time-consuming and limited by the number of leukemia cells in collected specimens.

Abnormal immunophenotype analysis by flow cytometry (FCM) has been widely used and extensively studied for the rapid diagnosis and monitoring of minimal residual disease (MRD) in hematologic malignancies, such as APL [5–9]. In the past decade, great effort has been made to identify APL characteristic immunophenotypic profiles for rapid and accurate diagnosis by FCM; however, no consensus has been established for the phenotypic profiles and corresponding analysis strategies. The characteristic immunophenotypic FCM features for APL have included a high side scatter (SSC), a typical consistent expression of cluster differentiation 117 (CD117), absence of HLA-DR, and absence or downregulation of CD34 in the context of myeloid antigen expression, such as myeloperoxidase (MPO), CD33 and CD13 [10–13]. Other common features include reduced expression of CD10, CD11a, CD11b, CD11c, CD45RO, and CD133 [1, 10, 14, 15]. However, these phenotypic profiles are not specific for differentiating APL from other non-APL AMLs [9–14].

It has been well documented that early treatment can significantly decrease the overall mortality associated with APL. Therefore, early confirmation of a morphologically suspicious APL by FCM is especially important and is theoretically easy to implement in clinical practice. Here, we describe an analysis strategy that combines an APL diagnostic immunophenotypic panel (ADI-panel) with a specific CD64 expression pattern. Our results demonstrate that the ADI-panel can rapidly distinguish APL from other AMLs with high diagnostic accuracy.

## RESULTS

### Immunophenotypic profiles of APL and APL-like-immunotypes in AML patients

**Figure 1:**
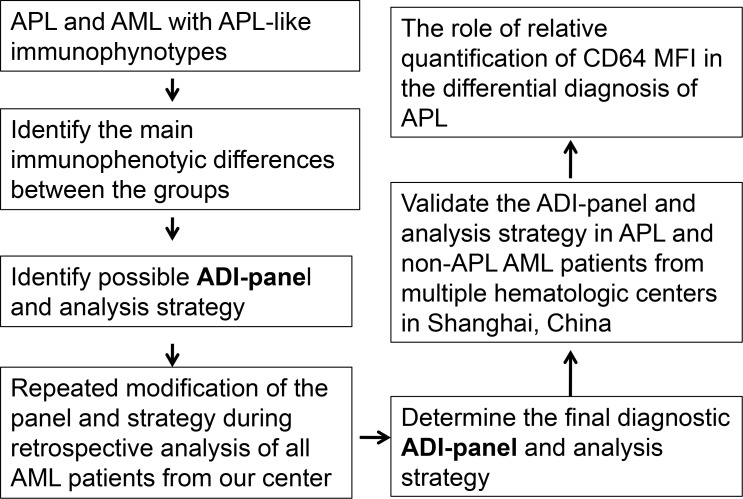
Flow chart for the identification and validation of the ADI-panel and the corresponding analysis strategy ADI-panel: APL diagnostic immunophenotypic panel; MFI: mean fluorescence intensity.

Identification of the ADI-panel and corresponding analysis strategy are shown in the flow chart in Figure 1. Non-APL AML cases with high expression of CD34, HLA-DR and/or CD117 are more easily differentiated from APL. In this study, we screened the potential unique immunophenotype of APL by selectively comparing homogenous AML patients whose leukemia cells did not express CD34 and HLA-DR, but expressed CD117 (Table 1), and who had no t(15;17) translocation or abnormal RARα fusion. Seventy-three patients identified as APL or AML with APL-like immunophenotypes were selected from 323 AML patients in Changhai Hospital, in which only 12.4% (40/323) were finally confirmed as having APL (Table 1). Most APL (87.5%, 35/40) patients also demonstrated a triad of absent or weak CD34 and HLA-DR, as well as consistent CD117 expression. The other 12.5% (5/40) of APL patients demonstrated positive CD34 and/or HLA-DR (Table 1) expression. There were 10.2% APL-like non-APL patients initially enrolled among the AML patients from Changhai Hospital (33/323, M1 = 5, M2 = 15, M4 = 12, and M6 = 1), accounting for 45.2% (33/73) of all the selected suspicious APL patients. When APL-like immunophenotypes were used to identify APL compared to MICM assay, the diagnostic performance demonstrated 87.5% sensitivity, 88.3% specificity, and 88.2% accuracy. The Kappa test (Kappa = 0.583, *P < 0.001*) showed a general consistency and the McNemar test revealed true differences between these two methods (*P < 0.001*, Supplementary Table 1).

**Table 1:** Clinical and immunophenotypic features of 73 enrolled APL and AML patients

	APL (*n* = 40)	AML (non-APL, *n* = 33)	*P*
	Positive Cases (%)	Positive Cases (%)	
CD117	40 (100%)	32 (97.0%)	0.452
MPO	40 (100%)	28 (84.8%)	0.016
CD13	40 (100%)	21 (63.6%)	< 0.001
CD33	40 (100%)	29 (87.9%)	0.038
CD64	40 (100%)	3 (9.1%)	< 0.001
CD11c	6 (15%)	21 (63.6%)	< 0.001
CD15	11 (27.5%)	8 (24.2%)	0.795
CD56	3 (7.5%)	7 (21.2%)	0.169
CD123	29 (72.5%)	23 (69.7%)	0.801
CD38	28 (70%)	25 (75.8%)	0.610
CD34	3 (7.5%)	0 (0%)	0.247
HLA-DR	2 (5%)	0 (0%)	0.498
CD2	3 (7.5%)	0 (0%)	0.247
CD14	0 (0%)	1 (3.03%)	0.452
CD11b	0 (0%)	4 (12.1%)	0.038
CD10	0 (0%)	0 (0%)	-
cCD79α	0 (0%)	0 (0%)	-
cCD3	0 (0%)	0 (0%)	-
CD4	0 (0%)	0 (0%)	-
CD16	0 (0%)	0 (0%)	-
CD7	0 (0%)	0 (0%)	-
CD19	0 (0%)	0 (0%)	-

Note: Seventy-three patients with APL-like immunophenotypes were selected from 323 AML patients in Changhai hospital between September 2011 and December 2014 in which only 54.8% were finally confirmed as APL.

**Figure 2:**
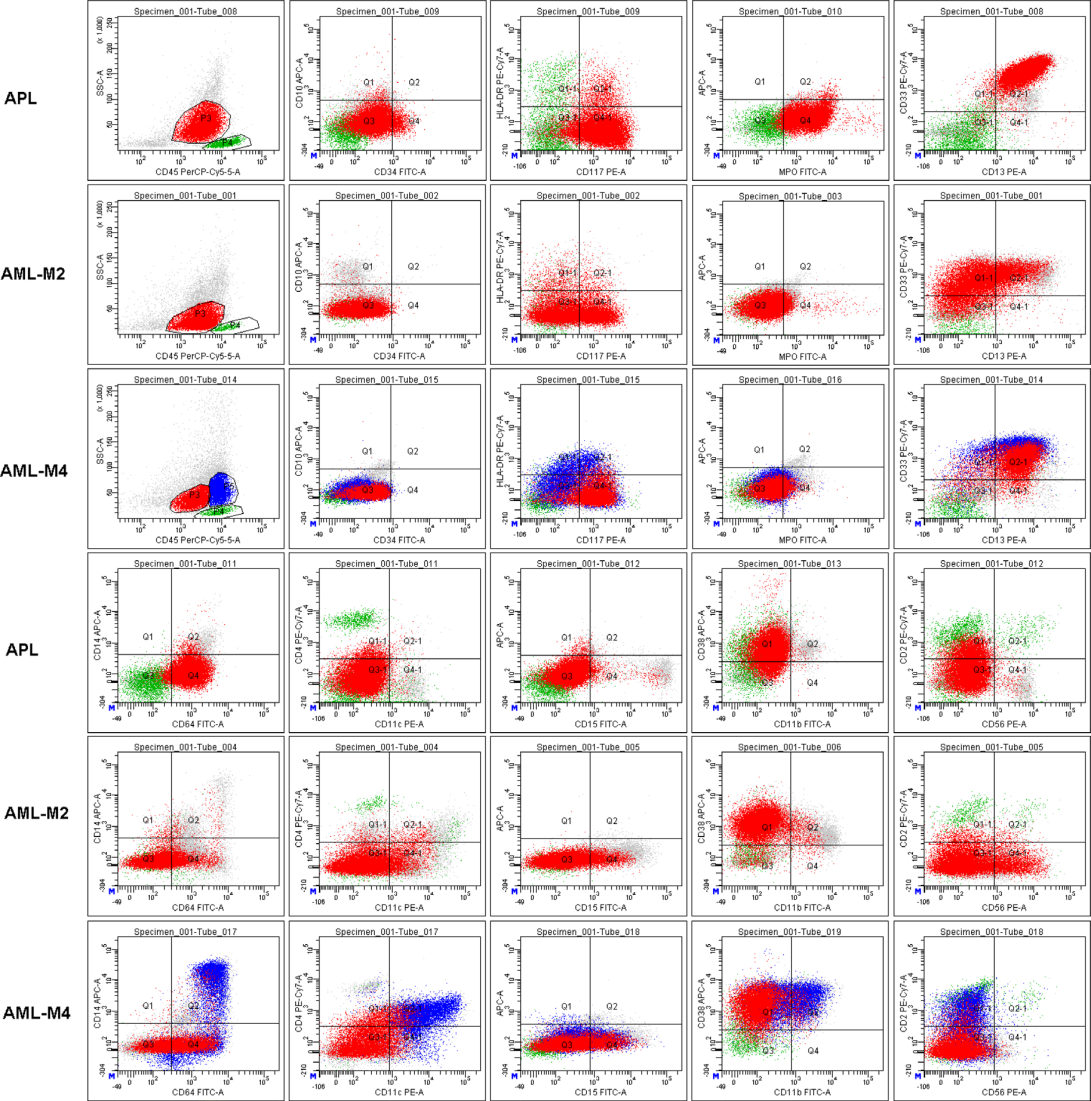
Representative characteristics of APL and non-APL AML patients (AML-M2 and M4) with indistinguishable immunophenotypes All leukemia cells (red cells and/or blue cells in AML-M4) in these representative patient samples expressed CD117, but had weak or no expression of CD34 and HLA-DR. Lymphocytes (green cells) were gated as an internal control for rational gating strategies. APL cells expressed MPO, CD13 and CD33 as determined by both positive rate and MFI. Almost all APL cells expressed CD64 with moderate MFI. However, only some of the leukemia cells in other AML patients expressed a heterogeneous lower intensity of CD64.

As shown in Table 1 and Figure 2, expression of MPO, CD13, CD33, CD64, CD11c, and CD11b were significantly different between APL and non-APL AML patients. The most obvious characteristic in APL leukemia cells was the positive expression of MPO (+, 100%), CD13 (+, 100%; and homogeneous mean fluorescent intensity (MFI)), CD33 (+, 100%; and homogeneous MFI), CD64 (dim, 100%; and homogeneous MFI), and negative expression of CD11b (0%) and CD10 (0%). Meanwhile, only some APL patients simultaneously expressed CD11c (15%). In sharp contrast, a few of the enrolled non-APL AML patients demonstrated equivalent intensity of CD64 (9.1%, *P* < 0.001) with less and heterogeneous positive expression of CD13 (63.6%, *P* < 0.001) and/or CD33 (87.9%, *P* = 0.038). However, CD11c was expressed in most non-APL AML patients (63.6%, *P* < 0.001), as determined by both positive rates and MFI (Figure 2).

### SSC of APL cells was significantly higher than non-APL AML cells

**Figure 3:**
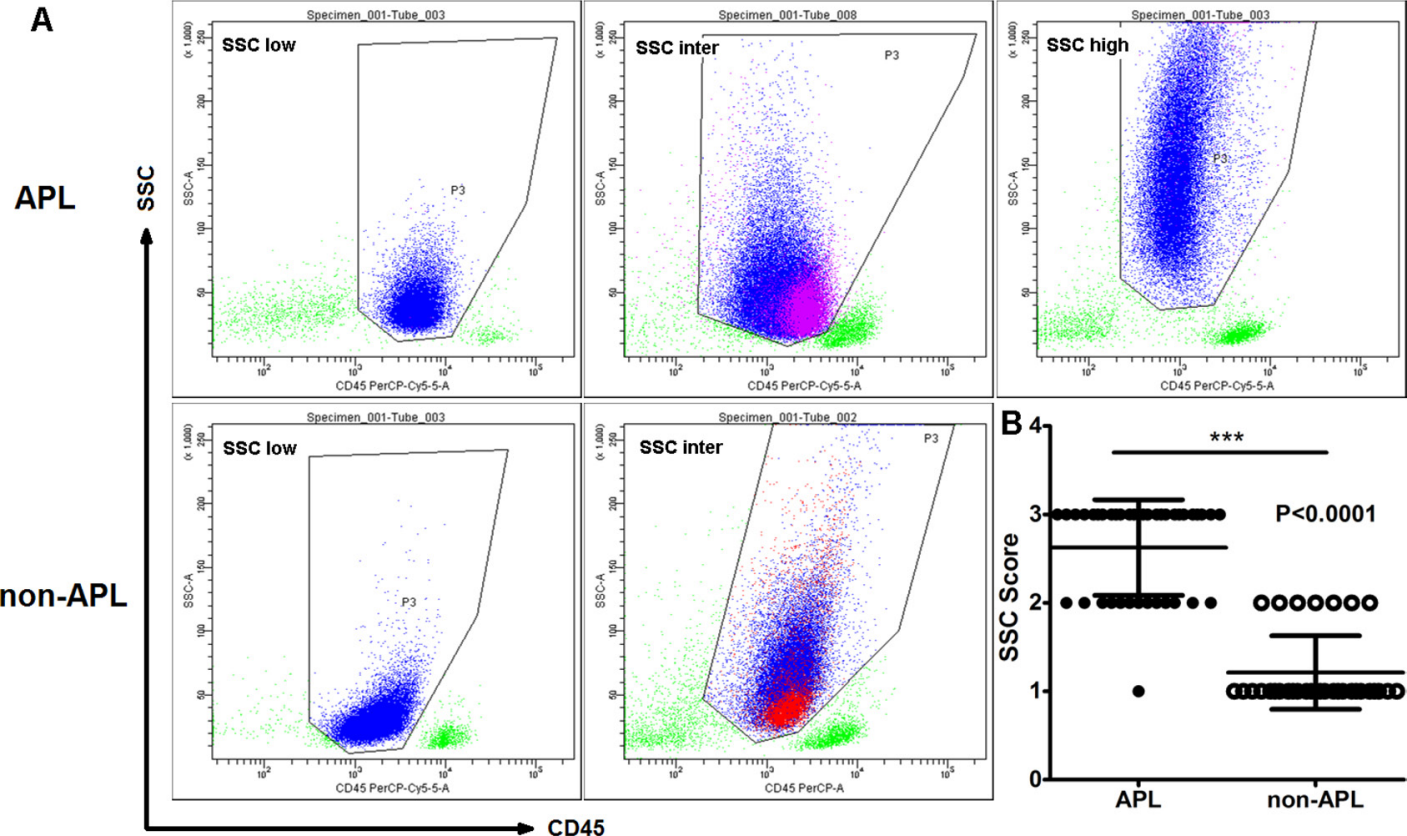
SSC signals in APL were higher compared to non-APL AML patients (**A**) Distribution of leukemia cells on the CD45/SSC scatter plots in APL and non-APL AML leukemia cells (blue cell population). CD34^+^ leukemia cells in APL patients were located in the CD45^dim^SSC^low^ area (purple cell population). CD117 was expressed only in some of the leukemia cells in the non-APL AML cells (red cell population), while most of the abnormal cells demonstrated low to intermediate SSC signals. (**B**) The SSC score of APL cells was significantly higher compared to leukemia cells in the non-APL AML patients (*P* < 0.001).

We next compared the SSC of leukemia cells between APL and non-APL AML groups. We defined the SSC signal as low (score = 1, main cell population located under 50 of the SSC-axis), intermediate (inter; score = 2, main cell populations located between 50 and 100 of the SSC-axis), or high (score = 3, main cell populations located higher than 100 of the SSC-axis). Using this designation, the photomultiplier tube (PMT) of SSC was considered suitable when the SSC signal of lymphocytes was located between 0 – 30 of the SSC-axis in the scatter plot of CD45-SSC (Figures 2 and 3). Nearly all leukemia cells in APL patients (except 1 case) were located in the CD45^dim^SSC^inter^ to CD45^dim^SSC^high^ area, while non-APL leukemia cells were more likely found in the CD45^dim^SSC^low^ to CD45^dim^SSC^inter^ area (Figure 3A). No CD45^dim^SSC^high^ cases were found in the non-APL AML patients (Figure 3B), which was in sharp contrast to the APL patients. Moreover, the CD34^+^ leukemia cells in the three APL patients were all located in the CD45^dim^SSC^low^ area (Figure 3A, purple cell population), and the remaining abnormal cells demonstrated intermediate SSC signals. In non-APL AML patients, CD117 was expressed in some of the leukemia cells that demonstrated intermediate SSC signals (Figure 3A, red cell population). These results demonstrate that both the physical signal and the CD markers of leukemia cells are significantly different between APL and non-APL AML patients, irrespective of their cytogenetic or morphological differences.

### An ADI-panel mainly including CD64, CD13, CD33, CD11c, and SSC signals can effectively distinguish APL from AML and normal bone marrow

**Figure 4:**
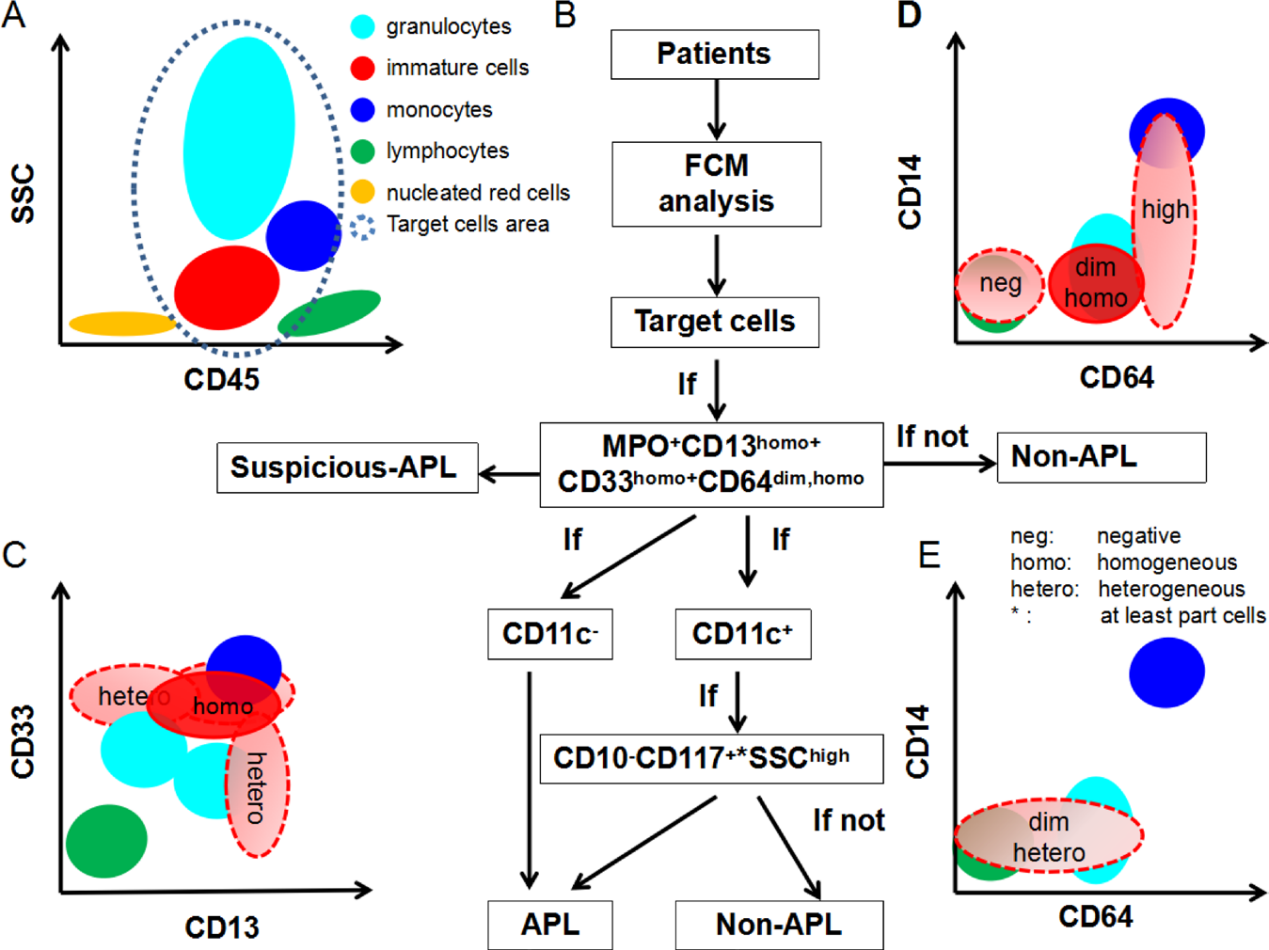
Scheme of ADI-panel and analysis strategy Distribution of primary normal or abnormal cell populations in BM, together with the interpretation of dim, homogeneous, heterogeneous expression of CD64, CD13 and CD33 are shown. (**A**) CD45-SSC schematic diagram for the distribution of common cell populations in BM. The target cell area in the gray-blue dashed line represents the concerned analysis area and is also the typical possible position of abnormal leukemia cells in APL and AML or other myeloid blood disease. (**B**) The diagnostic schematic flow-chart for APL. (**C**, **D**) Cell populations marked in red represent the nearly constant positions for the expression of CD13, CD33 and CD64 in APL cells compared to normal lymphocytes, monocytes and granulocytes. and (**E**) schematic diagram for the definition of negative, dim, high, homogeneous, or heterogeneous expression of CD13, CD33 and CD64. Light red populations with dashed borders demonstrate the typical expression patterns of CD64 in other AML cells. Homogeneous: uniformly positive expression with relatively minor CV of MFI; Heterogeneous: partial and continuous expression with relative major CV of MFI.

We next explored whether it is possible to efficiently distinguish APL patients from other AML patients using only FCM characteristics. As shown in Table 1 and Figure 2, CD64 and CD11c expression patterns and the characteristics of the SSC signal in the main abnormal cell subpopulations were the key differentiating factors between APL and those AML with similar APL immunophenotypes. Indeed, when the dim and homogeneous (homo) expression of CD64 and homogeneous expression of CD13 and CD33 were highlighted, we could provide an easy and valid ADI-panel and corresponding analysis strategy using only FCM (Figure 4). In this panel MPO was positive, and CD13 and CD33 were both positively and homogeneously expressed in nearly all APL cells. All leukemia cells located between the lymphocytes and monocytes with clear boundaries expressed dim and homogeneous CD64 (Figures 2 and 4D, CD64^dim and homo^). APL diagnosis was established if negative expression of CD11c was further confirmed. In CD11c^+^ patients, positive CD117 (at least in some of the leukemia cells), negative CD10, and inter to high SSC signal were necessary and sufficient for APL diagnosis (Figure 4B). Negative expression of CD4, CD14 and CD16 further confirmed, but were not required for, APL diagnosis.

We then validated our established strategy in the enrolled 40 APL and 33 non-APL AML patients. The results showed that the MPO^+^CD13^homo+^CD33^homo+^CD64^dim and homo^ CD11c^−^ or MPO^+^CD13^homo+^CD33^homo+^CD64^dim and homo^CD11c^+^CD117^part∼+^SSC^inter-high^ CD10^−^ panel efficiently identified APL with 100% sensitivity, specificity and accuracy among those non-APL patients with a CD34^−^HLA-DR^−^CD117^+^ immunopenotye. In addition, high sensitivity (99.0%), specificity (99.2%), and accuracy (99.16%) were obtained when these two patterns were applied to diagnose 834 primary adult AML patients, including 205 APL patients and 629 other M1, M2 or M4 - M7 patients from the two main hematology departments in Shanghai (Table 2). In this case, the Kappa test (Kappa = 0.977, *P* < 0.001) showed great consistency and the McNemar test revealed no difference between the ADI-panel and the MICM assay (*P = 0.453*, Table 2).

**Table 2:** ADI-Panels accurately recognize APL patients from AML patients in our multi-centers study

ADI-Panel	MICM		Total
	APL	Non-APL	
APL	200	5	205
Non-APL	2	627	629
Total	202	632	834

Note: ADI-Panels, APL diagnostic immunophenotypic panel; MICM, morphology, immunology, cytology, and molecular biology; Data comparisons were performed using McNemar (*P* = *0.453*) and Kappa tests (Kappa = 0.977, *P* < *0.001*).

In this study, CD11b but not CD11c was routinely detected in the patients from Ruijin Hospital. Since the expression characteristics of CD11b are similar to CD11c during normal neutrophilic maturation, we used CD11b as a substitute for CD11c. CD10 was also not routinely included in the Ruijin AML panel. Therefore, CD15 (a maturation marker normally expressed on neutrophils) was used as a substitute for CD10 to help differentiate APL cells from neutrophils when CD11b was positively expressed. However, CD11b expression was lower than CD11c in APL cases from Changhai Hospital, as shown in Table 1, but CD11b expression was 8.1% (14/173) in APL cases from the Ruijin Hospital. CD15 was expressed in both APL and non-APL cells. Therefore, the substitution of CD11c and CD10 with CD11b and CD15 may partially affect the performance of the ADI-panel.

Another 200 randomly selected bone marrow samples from patients with lymphoma in Changhai Hospital were used as a “normal bone marrow control”, in which no lymphoma cells were identified. These samples were used to evaluate the differentiation potential of our ADI-panel for reorganizing APL samples with normal granule cells and monocytes in“normal” bone marrow, since the APL cells always appeared in the similar area in CD45/SCC dot-plot pictures. In these cases, we gated and compared all granule cells (including mature or immature myeloid cells) and monocytes. In this analysis, we report 100% specificity and 100% negative prediction using the ADI-panel (data not shown).

### ADI-panel accurately recognizes atypical APL patients

**Figure 5:**
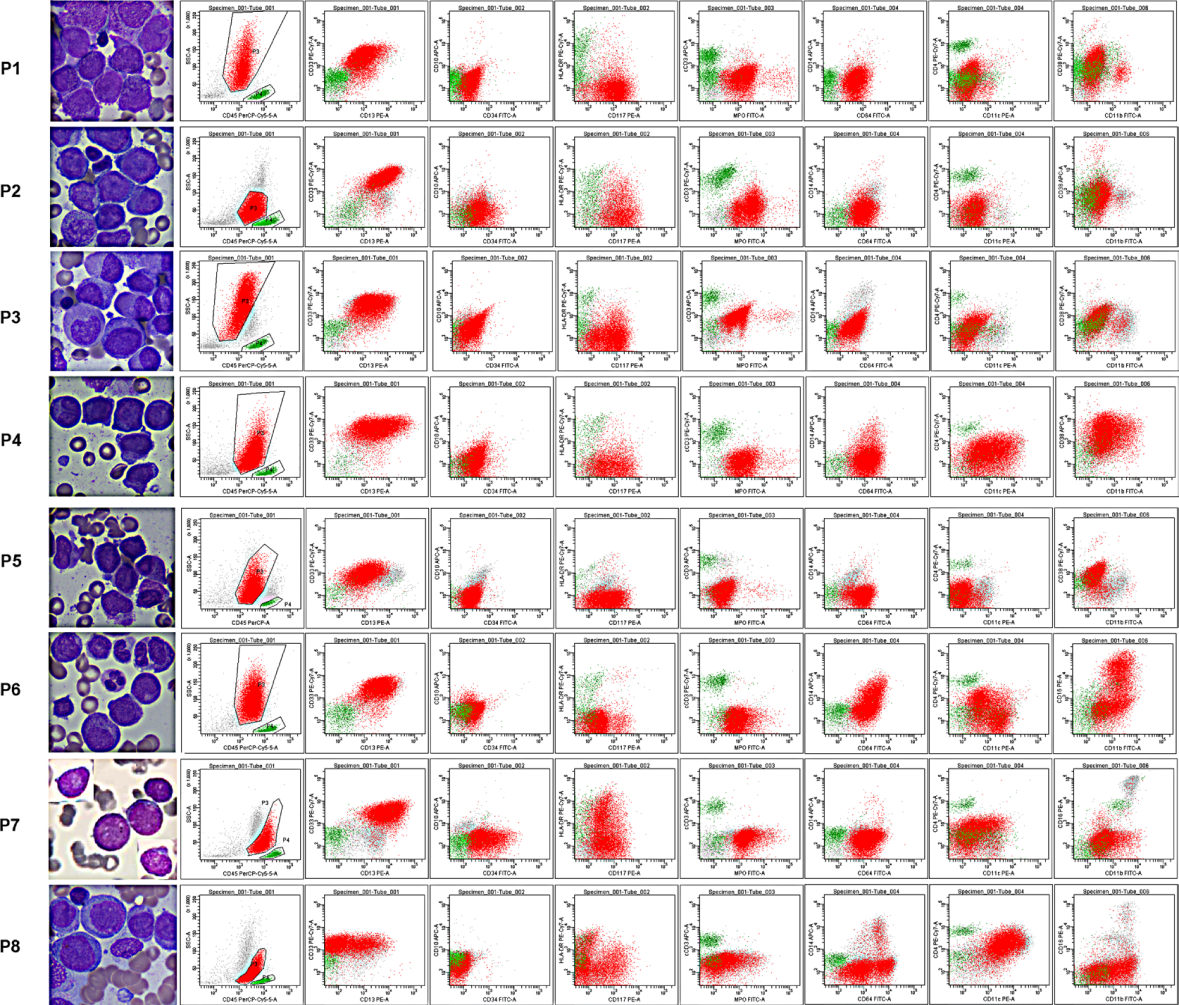
Morphology and immunophenotypes of atypical APL patients The primary laboratory characteristics of atypical APL patients are listed in Table 3. Patients (P) 1−7 were APL patients with atypical morphology (P 2, 3, 7) or with other rare translocations within chromosome 17 (P 1, 3−7). Patient 8 was an AML-M5 patient and was used as a morphologic and immunophenotypic control for macroscopic comparison of atypical morphology and a better understanding of the expression patterns of the selected CD markers and SSC signals. Lymphocytes are colored in green and leukemia cells in red.

A subset of AML cases, often with morphological features resembling APL, show variant translocations involving RARa. These variant fusion partners include promyelocytic leukemia zinc finger gene (PLZF) at 11q23, the nuclear matrix associated gene (NUMA1) at 11q13, the nucleophosmin gene (NPM1) at 5q35, and STAT5B at 17q11.2 [4]. Other APL patients may have abnormal translocation of chromosomes other than the classic t(15;17). We tested our established ADI-panel and diagnosis strategy in APL patients from Changhai Hospital, who presented with atypical morphologic variants and/or variant translocations involving RARα other than PML-RARα. The main clinical characteristics of these patients are listed in Table 3, and the typical scatter plots and morphologic pictures are compared in Figure 5. Patient 8 was an AML-M5 and was used for morphologic comparison with no or hypogranular cytoplasm (Patient 2 and 3, respectively), for similar cell distribution comparison in CD45 and scatter plots (Patient 7), and for the expression pattern comparison of all analyzed CD markers (Patients 1−7). Although atypical morphologic characteristics, rare patterns of chromosome translocation, and variant fusion partners of RARα have complicated APL diagnosis for a long time, our ADI-panel efficiently identified all of these patients as having atypical APL. CD11b, which was thought to be absolutely negatively expressed on APL cells and has been used for differentiating APL from other AML patients [1], was found to be expressed in two of our APL patients (Patients 4 and 6). Expression of CD14, CD4, CD16, CD34, and HLA-DR occasionally appeared in different APL patients and were thus not reliable markers for the diagnosis or exclusion of APL. Patient 7, who died one month after a non-APL AML diagnosis in another hospital because of serious diffuse intravascular coagulation (DIC), demonstrated hypocellular BM and atypical APL morphology. Expression of CD34, CD117, HLA-DR, CD11b, and CD11c further complicated diagnosis in Patient 7, but our ADI-panel suggested APL diagnosis before the existence of PML-RAR*α*, and t(7;15;17) translocation was further confirmed by PCR, karyotyping and FISH. These data demonstrate that our established ADI-panel can also accurately recognize atypical APL patients.

**Table 3:** Laboratory characteristics of APL patients with atypical morphologic variants and/or with abnormal RARα fusion genes other than PML-RARα and/or abnormal karotyping other than t(15; 17)

	Diagnosis	Morphology	Fusion gene (PCR)	Karyotyping	FISH: Fluorescence mode
Patient 1	APL	typical large and numerous cytoplasmic granules	PML-RARα-L	46,xy,ins(15;17)	PML-RARα: 1Y2R1G
Patient 2	APL	non or hypogranular cytoplasm	PML-RARα-S	46,xx,t(15;17)	PML-RARα: 2Y1R1G
Patient 3	APL	myelocytes and metamyelocytes with reducing cytoplasmic granules	NPM-RARα	46,xy,t(5;17),7p-,-16 [8]/46,idem,+20 [5]	RARα:1Y1R1G
Patient 4	APL	typical large and numerous cytoplasmic granules	PML-RARα-S	46,xx,t(15;2;17)	PML-RARα:1Y2R2G
Patient 5	APL	typical large and numerous cytoplasmic granules	PML-RARα-L	46,xy,t(15;17;17)(q22;q25;q21)	PML-RARα:1Y2R2G
Patient 6	APL	typical large and numerous cytoplasmic granules	PML-RARα-L	46,xx,t(15;17)	PML-RARα:1Y2R2G
Patient 7	APL	hypocellular BM and hypogranular cytoplasm	PML-RARα	46,xx,t(7;15;17)	PML-RARα:1Y2R2G
Patient 8	AML-M5	no cytoplasmic granules	ND	46,xx	ND

Note: ND, not detected.

### CD64 MFI expression is a diagnostic marker for APL

**Figure 6:**
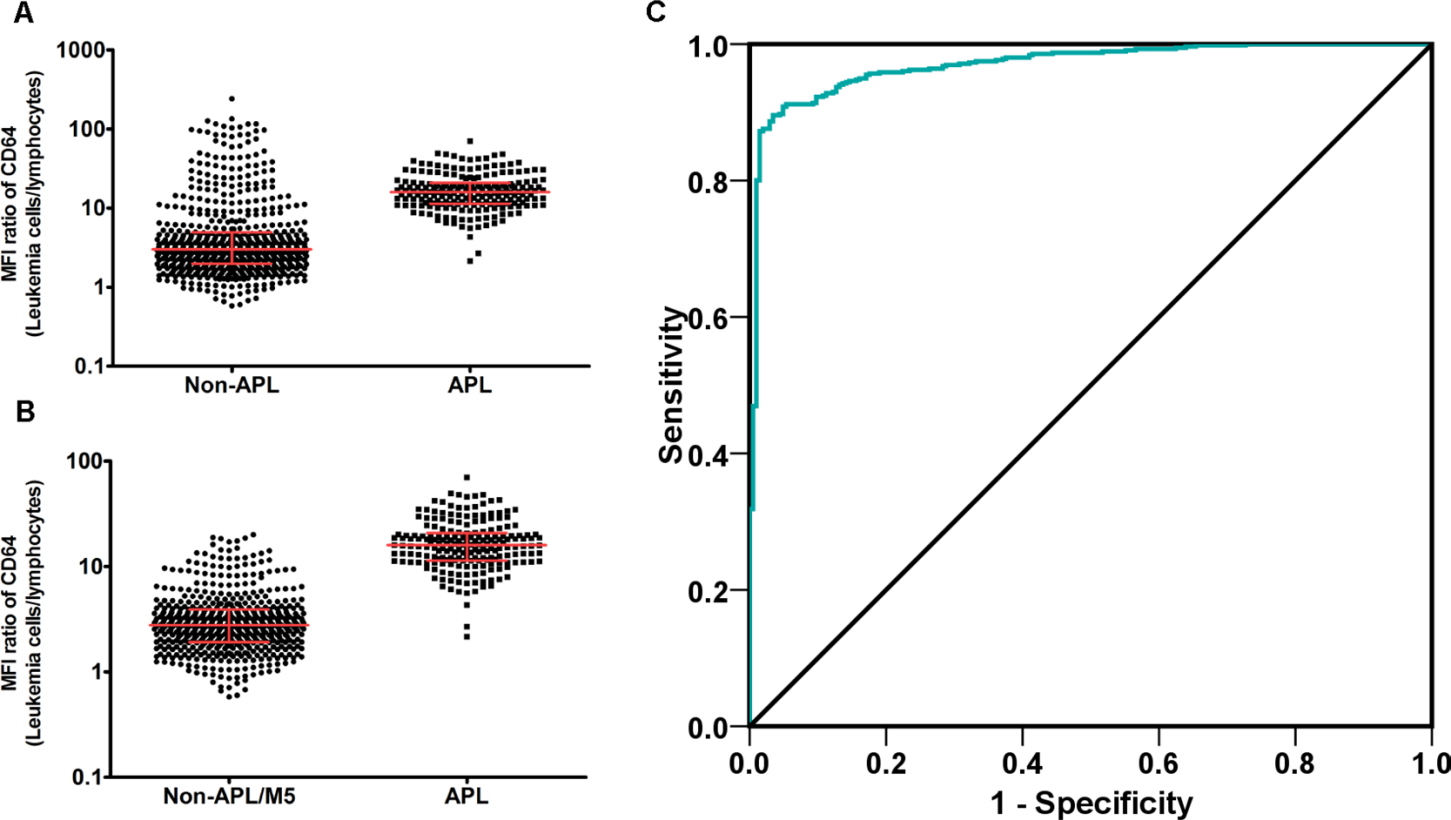
MFI ratio of CD64 (Leukemia cells/Lymphocytes) is an efficient diagnostic marker of APL MFI ratio of CD64 expression was defined as: Ratio value = CD64 MFI of Leukemia cells/CD64 MFI of Lymphocytes. (**A**) MFI ratio of CD64 in 629 non-APL AML patients was compared to 205 APL patients. Line and bars stand for median with interquartile range, non-APL (3.01, 1.99−4.97); APL (15.91, 11.37−20.80), *P* < *0.0001*. (**B**) MFI ratio of CD64 in 593 non-APL/M5 AML patients was compared to 205 APL patients. Line and bars stand for median with interquartile range, non-APL (2.785, 1.910−3.895); APL (15.91, 11.37−20.80), *P* < *0.0001*. (**C**) ROC of CD64 MFI ratio in APL diagnosis. Area under curve (AUC) was 0.970 with an 95% CI: 0.958−0.982 (*P* < *0.0001*).

To eliminate the potential subjective discrepancy of using CD64 in the differential diagnosis of APL for different hematology centers, we next quantified CD64 by obtaining a ratio of CD64 MFI on APL cells to that of lymphocytes. In this way, the different antibody clones and different fluorescein of CD64 antibodies might be relatively standardized and the rules we set might be accepted by more hematology centers. As shown in Figure 6A, among the 834 AML patients, the MFI ratios of CD64 (Leukemia cells/Lymphocytes) in APL patients (mean, 95% confidence interval (CI): 18.30, 16.88−19.71) were significantly higher than those in non-APL patients (mean, 95% CI: 9.32, 7.68−11.0). Since CD64 was highly expressed in AML-M5 patients (CD64 ratio of AML-M5 was also higher compared to APL in our cohort; Figure 6A, high value dots), we again compared APL patients with non-APL/M5 AML patients in this cohort by removing the AML-M5 patients whose diagnosis was confirmed primarily by morphology. More discrimination was identified with lower and more homogeneous MFI ratios of CD64 in non-APL/M5 patients (mean, 95% CI: 4.92, 4.42−5.42) compared to APL patients (Figure 6B, 205 APL, 556 non-APL/M5 patients). Thus, the typical characteristic difference in MFI ratios of CD64 in APL cells was between non-APL/AML-M5 and AML-M5 cases. Therefore, if all of the data in Figure 6A are included in a ROC curve analysis, the diagnostic performance of CD64 ratio will be inevitably artificially underestimated. It is therefore more objective to evaluate these APL data separately as either non-APL/AML-M5 or AML-M5. Since AML-M5 was easy differentiated from APL by relative high value of CD64 ratio, we therefore drew ROC curves to evaluate the diagnosis performance of the MFI ratio of the CD64 marker alone to differentiate APL from non-APL/M5 patients. The area under the curve (AUC) was 0.970 with a 95% CI: 0.958−0.982 (*P* < 0.0001), which demonstrates a near perfect performance of relative quantification of CD64 marker in differentiating APL from non-APL/M5 patients.

## DISCUSSION

The expression characteristics of the lineage cluster CD markers in hematopoietic cells have been successfully and widely applied for the analysis and isolation of different blood cells with multicolor FCM [16]. Myeloblasts are positive for CD34, HLA-DR, CD117, CD38, CD13, and CD33. CD34 and CD117 are expressed in all hematopoietic precursors, including early myeloblasts. CD13 appears before the acquisition of CD33. Neutrophilic maturation from blasts through promyelocytes, myelocytes, metamyelocytes, and neutrophils is characterized by loss of CD34 and HLA-DR at the promyelocytic stage, with loss of CD117, and acquisition of CD11b and CD11c at the myelocytic stage. CD11b intensity increases as cells mature to late myelocytes and metamyelocytes. CD64 is expressed by promyelocytes through metamyelocytes. CD13 and CD33 are expressed at all stages of maturation, but with a slightly different variation in expression intensity. Metamyelocytes start to express CD10 and CD16 as the cells progress to mature neutrophils. Segmented neutrophils display higher expression of CD11b, CD11c, CD10, CD16, and CD15. In general, normal promyelocytes positively express CD117, CD13, CD33, CD64, and MPO, but have negative expression of CD34, HLA-DR, CD11b, CD11c, CD10, and CD16 [16, 17].

APL is an AML with abnormal accumulation of promyelocytes. APL is mainly caused by the formation of abnormal *PML-RARα* fusion gene resulting from the translocation of chromosome 15 and 17 breakages and the reunion of bands 15q22 and 17q12. Most APL cells also possess the immnunophenotypic profiles of normal promyelocytes, including positive expression of CD117, CD64, CD33, and CD13, but are negative for CD34, HLA-DR, CD11b, CD11c, and CD10 [10–15]. Accordingly, FCM immunophenotypic analysis has been widely used for differential diagnosis of leukemia and has greatly facilitated the prompt diagnosis of APL. However, no accordance of specific immunophenotypic characteristics of APL, which could be used for a definitive APL diagnosis without cytological changes, has been established.

We hypothesize that a combination of specific immunophenotype markers might effectively distinguish APL from other types of AML without reference to molecular cytological changes. Consistent with previous reports, we demonstrated that CD117^+^ HLA-DR^−^CD34^−^ was a classic immunophenotype pattern for APL, but showed that it had nearly no role in diagnosis. Most importantly, we are the first, to our knowledge, to report that the expression pattern of CD64 is a critical determinant in APL diagnosis. Intriguingly, we revealed that MPO^+^CD13^homo+^ CD33^homo+^CD64^dim and homo^ constituted the basic expression profiles for nearly all APL immunodiagnoses. The simultaneous dim and homogeneous expression of CD64 was the mandatory standard in this combination, and APL diagnosis should be excluded if any of the four markers is unsatisfied. It is important to note that CD64 is usually expressed by promyelocytes through metamyelocytes, and CD64 expression intensity is always lower than that in monocytes [16]. Moreover, CD64 expression on APL cells is also dimly expressed compared to acute monocytic leukemia cells, which might lead to false negative APL diagnoses in patients [18]. This might also be the main reason for the high variation of CD64 expression in APL in the published data (17.5−100%) [1, 10, 17, 19, 20]. CD64 was 100% positively expressed in all of our clinical APL patients, suggesting that APL should be excluded in the absence of CD64 expression.

The expression patterns of CD11c and/or CD117 and the characteristics of SSC signal help differentiate APL from non-APL AML, regardless of the expression of other molecules such as CD34, HLA-DR, and CD2. Dong *et al*. suggested that CD11c and CD11b are negatively expressed in APL patients and should be included in the diagnostic immunophenotypic panel of APL patients [1]. However, both in our retrospective and perspective analyses of APL patients, we identified expression of CD11b and CD11c on leukemia cells. CD11c expression first appears on granulocytes at the end of promyelocytes and through the myelocytic stage during the development of normal hematopoietic cells [16, 17]. Therefore, although there is an absence of CD11c expression in most APL patients, the expression of CD11c on APL might not be exclusive, especially when the leukemia cells demonstrate more mature states. In APL patients with CD11c expression, the leukemia cells might be in a more mature state, closer to the phenotype of normal myelocytes, that possess higher SSC signal compared to normal myeloblasts and promyelocytes. Therefore, high SSC signal should be added as a necessary condition. Indeed, our data demonstrated that a high SSC signal was nearly an essential and sufficient marker for the immunodiagnosis of APL when CD11c was expressed. CD10, meanwhile, was found to be negatively expressed in all of our enrolled APL patients, and thus was incorporated into the ADI-panel to ensure diagnostic specificity. Taken together, the characteristic combination of MPO^+^CD13 ^homo+^CD33 ^homo+^CD64 ^dim and homo^ CD11c^−^ markers or MPO^+^CD117^+^CD13^homo+^CD33 ^homo +^ CD64^dim and homo^ CD11c^+^CD10^−^SSC^inter∼high^ markers accurately and efficiently recognized APL in our laboratory. Further prospective validation using our or other hospital patient samples revealed a nearly 100% accuracy of this ADI-panel and analysis strategy. The substitution of CD11c with CD11b and CD15 in other hematology centers, where CD11c is not routinely detected, might slightly limit the accuracy of the ADI-panel. It has been reported that simultaneous expression of CD34 and CD2 in leukemia cells is indicative of an immature immunophenotype of APL and is thought to be associated with the internal tandem duplication of the FLT3 gene (FLT3/ITD) [21, 22]; however, we found that these two markers played no role in the diagnosis of APL in our APL specific immunophenotypic combinations.

Nevertheless, the reliability of our ADI-panel might vary greatly in different laboratories due to the inadequate standardization of the protocol, the diverse brands of FCM instruments, the wide variety of available antibodies against the same marker, many alternative fluoresceins with different fluorescence intensity, and most importantly, the variation in experienced operators and reporters [8, 19, 23–25]. Nevertheless, the current comparability of the FCM results is far below other clinical routine blood or biochemical testing, especially in leukemia diagnosis. Thus, this may explain the concordance of the main APL phenotypic characteristics (MPO^+^CD117^+^HLA-DR^−^CD34^−^CD13^+^CD33^+^) with the inconsistency of their positive rate and/or expression MFI, and even greater difference in the mature markers (CD64, CD11c, CD11b, CD10, SSC signal and etc.) in the literature [10, 17–20]. Based on our results, together with the carefully selected clinical control specimens possessing more complex immunophenotypes, we suggest a more characteristic immunophenotypic expression profile of APL with an improved specificity for early diagnosis of APL using only FCM. However, when using our conclusive ADI-panel and the corresponding analysis strategy, one must first verify our results in his/her own experimental system with previously diagnosed APL patients. Application of our ADI-panel must be tested in conjunction with molecular genetic evidence and optimized to establish standards of positive or negative, as well as bright or dim, expression patterns. In order to further evaluate the value of CD64 in APL diagnosis and to use this marker more conveniently and objectively, we obtained a ratio of CD64 MFI on APL cells to that of lymphocytes which could be easily repeated in each FCM department. Most intriguingly, the MFI ratio of CD64 in APL cells always fell in the middle of those non-APL/M5 AML and AML-M5 patients with remarkable discrepancies, revealing an extremely perfect diagnosis performance in identifying APL with this single MFI ratio.

It should be noted that our ADI-panel might miss rare APL patients who lack expression of CD13 and/or CD33, similar to what has been reported in a previous study [1]. The clinical data from the previous study should be extensively confirmed, since part of their conclusions are not consistent with our and other studies, including the negative expression of CD11b and CD11c in APL patients and the expression of CD64 in only some of the APL patients. Furthermore, FCM is suggested for follow-up of APL patients during treatment because of the common change in morphological features of the leukemia cells at the time of relapse, which could result in misdiagnosis of a different type of AML [15, 26]. Therefore, a comprehensive approach with emphasis on combined morphological, immunophenotypic and cytogenetic analyses is important for the diagnosis and appropriate treatment of relapsed APL.

In summary, the current study suggests a characteristic immunophenotypic profile that can facilitate a nearly 100% accurate and rapid diagnosis of APL with only flow cytometry. A single marker of MFI ratio, CD64, can be easily obtained and efficiently distinguish APL from non-APL AML patients. Application of our ADI-panel will help shorten the diagnosis duration and improve personalized treatment for APL, a life-threatening disease with very rapid progression.

## MATERIALS AND METHODS

### Ethics statement

This study was approved by the Changhai Hospital Institutional Review Board (Shanghai, China) in accordance with the Declaration of Helsinki.

### Patients

APL and AML patient diagnoses were based on criteria established by the “World Health Organization Classification of Tumors of Hematopoietic and Lymphoid Tissues (2008)” [4]. APL was confirmed in all patients by real time quantitative RT-PCR, chromosome karyotyping, and/or FISH, as described in the following sections.

At least one diagnostic bone marrow (BM) aspirate sample from each patient was submitted for flow cytometric immunophenotyping, RT-PCR, and cytogenetic analysis. FISH was used to screen for the PML-RARα fusion gene in all patients upon primary diagnosis. A total of 73 patients was initially enrolled for screening of our specific ADI-panel. Patients were selected from 323 AML patients hospitalized in the Institute of Hematology of Changhai Hospital between September 2011 and December 2014. There were 40 APL patients (18 male; 22 female) with a median age of 42.5 years (range, 15−69 years) and 33 AML patients with CD34^−^CD117^+^HLA-DR^−^ APL-like immnophenotypes (14 male; 19 female) with a median age of 49 years (range, 33−80 years). Another 834 primary patients who were diagnosed with AML (205 APL patients (98 male, 107 female; median age of 48.5 years range from 15 to 81 years); 629 other M1, M2 or M4 - M7 patients (298 male, 331 female; a median age of 52 years range from 17 to 88 years) from the participating hematology departments between January 2012 and November 2016 were further enrolled for validating the established ADI-panel and the corresponding analysis strategy. All APL was confirmed by the existence of PML-RARα, NPM-RARα, PLZF-RARα, or t(15;17) with RT-PCR, and/or conventional karyotyping, and/or FISH. The ADI-panel specificity for differentiating mature granulocytes or monocytes was also evaluated in another 200 randomly selected BM samples from patients with iron deficient anemia or lymphoma without BM infiltrated in Changhai Hospital (92 male; 108 female; median age of 41 years (range, 16−83 years)).

### Flow cytometric immunophenotyping

Flow cytometric immunophenotyping was performed using a panel of antibodies designed for AML. The detailed information of monoclonal antibodies (mAbs), reagents and panels used at diagnosis are shown in Supplementary Tables 2 and 3. Flow cytometric immunophenotyping was performed in 5-color (Changhai Hospital) and 10-color (Ruijin Hospital) combinations. Fresh heparinized BM samples were collected at diagnosis. After incubation with reagent cocktails for 15 min at room temperature, erythrocytes were lysed with BD (Becton Dickinson & Company) FACS^TM^ lysing solution (BD Biosciences; San José, CA, USA) or Ammonium Chloride (NH_4_Cl) based lysing solution (BD 555899) using a standard lyse/wash technique. For cytoplasmic antigens such as cMPO, cCD3 and cCD79a, the samples were processed using the Fix-and-Perm kit (BC, A07803) according to the manufacturer's guidelines. All antibodies were obtained from BD Biosciences or Beckman-Coulter (Marseille, France). Data were acquired and analyzed by flow cytometry FACSAria II with Diva software (BD, San José, CA, USA) and NAVIOS^TM^ with Kaluza software (BC, Marseille, France) in Changhai and Ruijin Hospitals. Cell surface antigen expression was considered positive if greater than 20% of the analyzed events were stained, while a cutoff of greater than 10% was set for cytoplasmic antigens. Immunological criteria for lineage affiliation and subtype were applied according to the NCCN 2016 recommendations.

### Conventional karyotyping and FISH

Chromosome analyses (R-banding) were performed on diagnostic BM samples that were prepared from stimulated BM aspirate cultures using standard techniques. Twenty metaphases were analyzed and reported using the International System for Human Cytogenetic Nomenclature. FISH for PML-RARα was performed on interphase nuclei using the Vysis LSI PML-RARα ES, dual-color, translocation, locus-specific probe (Abbott Molecular; Des Plaines, IL, USA). A cutoff value of 1.0% defined a positive result for PML-RARα.

### Real time quantitative RT-PCR assay

STAT5b/p1p1L/PRK/NUMA/NPM-RARα, PML-RARα-L, PML-RARα-S, and PLZF-RARα fusion transcript levels were quantified by RT-PCR. RNA was extracted from BM samples using Trizol reagent (Life Technologies; Grand Island, NY, USA) according to the manufacturer's instructions. Reverse transcription was performed on total RNA (1 ng) using random hexamers and SuperScript II reverse transcriptase (Life Technologies; Grand Island, NY, USA). Quantitative real-time PCR was performed using a Tagman probe (Shanghai Yuanqi Bio-Pharmaceutical CO., LTD) and analyzed using an ABI 7500PCR System (Applied Biosystems, Foster City, CA, USA). Primer sequences for the reference gene, ABL Proto-Oncogene 1 (ABL), and the genes of interest are listed in Supplementary Table 4. The typical PCR thermocycler profile was as follows: initial step at 95°C for 5 min followed by a second step at 94°C for 15 s and 60°C for 60 s for 40 cycles. The quantification standard curve was used to quantify the target mRNA expression and the level of target mRNA was also normalized to ABL.

### Statistical analysis

All statistical calculations were performed with SPSS software (SPSS 15.0; Chicago, IL, USA) or GraphPad Prism 5. Pair-wise comparisons between characteristics of patients were performed using McNemar and Kappa tests. The Fisher's exact test was used for non-paired categorical variables. Mann Whitney Test was used for the comparison of two groups of SSC score and relative CD64 ratio.

## SUPPLEMENTARY MATERIALS TABLES


