# Supplementary material for: Flow cytometric analysis of CD64 expression pattern and density in the diagnosis of acute promyelocytic leukemia: a multi-center study in Shanghai, China
Source: Oncotarget. 2017 Sep 11;8(46):80625–37. doi: 10.18632/oncotarget.20814 (PMC5655225; doi:10.18632/oncotarget.20814)
Supplement: Supplementary file 1 [file oncotarget-08-80625-s001.pdf]

Flow cytometric analysis of CD64 expression pattern and density in the diagnosis of acute promyelocytic leukemia: a multi-center study in Shanghai, China

SUPPLEMENTARY MATERIALS

Supplementary Table 1: APL-like immunophenotype recognizes APL patients from AML patients with low diagnostic performance

APL-like immunophenotype	MICM		Total
	APL	Non-APL	
APL	35	33	78
Non-APL	5	250	255
Total	40	283	323

Note: APL-like immunophenotype, expression pattern including CD117 + /CD34-/HLADR-; ADI-Panels, APL diagnostic immunophenotypic panel; MICM, morphology, immunology, cytology, and molecular biology. Data comparisons were performed using McNemar ($P < 0.001$) and Kappa tests (Kappa = 0.583, $P < 0.001$).

Supplementary Table 2: Information and panels of mAbs for diagnosis of AML in Changhai Hospital

mAb	mAb clone	Fluorochrome	Catalogue number	Source
CD2	39C1.5	PE-CY7	A21689	BC (Marseille, France)
CD4	SFCI12T4D11	PE-CY7	737660	BC (Marseille, France)
CD7	4H9	FITC	347483	BD (San Diego, France)
CD11b	Bear1	FITC	IM0530U	BC (Marseille, France)
CD13	L138	PE	347837	BD (San Diego, USA)
CD13	L138	PE-CY7	338425	BD (San Diego, USA)
CD14	RM052	APC	IM2580	BC (Marseille, France)
CD15	MMA	FITC	332778	BD (San José, CA, USA)
CD16	B73.1	PE	347617	BD (San José, CA, USA)
CD19	SJ25C1	APC	A78837	BD (San José, CA, USA)
CD33	D3HL60.251	PE-Cy7	A54824	BC (Marseille, France)
CD34	8G12	FITC	IM2709U	BD (San José, CA, USA)
CD34	8G12	APC	345807	BD (San José, CA, USA)
CD38	HB7	APC	551400	BD (San José, CA, USA)
CD45	2D1	PERCP	347464	BD (San José, CA, USA)
CD56	N901	PE-CY7	A21692	BC (Marseille, France)
CD64	22	FITC	IM1604U	BC (Marseille, France)
CD117	04D2D1	PE	IM2732	BC (Marseille, France)
CD123	7G3	APC	560087	BD Pharmingen (San Diego, USA)
HLA-DR	L243	PE-Cy7	335795	BD (San José, CA, USA)
cCD3	UCHT1	PE-CY7	737657	BC (Marseille, France)
CD79a	HM47	PE	IM2221	BC (Marseille, France)
MPO	CLB-MPO-1	FITC	IM1874U	BC (Marseille, France)
Fix-and-Perm	-	-	A07803	BC (Marseille, France)
Lysing Solution			349202	BD (San José, CA, France)

Panels for diagnosis of AML:
Tube1 CD7-FITC / CD13-PE / CD45-PERCP / CD33-PE-CY7 / CD19-APC
Tube2 CD34-FITC / CD117-PE / CD45-PERCP / HLA-DR-PE-CY7 / CD10-APC
Tube3 MPO-FITC / cCD79a -PE / CD45-PERCP / cCD3-PE-CY7
Tube4 CD64-FITC / CD11c-PE / CD45-PERCP / CD4-PE-CY7 / CD14-APC
Tube5 CD15-FITC / CD56-PE / CD45-PERCP / CD2-PE-CY7 / CD123-APC
Tube6 CD11b-FITC / CD16-PE / CD45-PERCP / CD13-PE-CY7 / CD38-APC

Notes: BD:Becton Dickinson & Company; BC: Beckman-Coulter

Supplementary Table 3: Information and panels of mAbs for diagnosis of AML in Ruijin Hospital

mAb	mAb clone	Fluorochrome	Catalogue number	Source
CD2	TS1/8	PB	309216	Biolegend (San Diego, USA)
CD4	PRA-T4	APC eFluor 780	47-0049-42	eBioscience (San Diego, USA)
CD7	8H8.1	PE	IM1429U	BC (Marseille, France)
CD11b	Bear1	FITC	IM0530U	BC (Miami, USA)
CD13	WM15	APC	301706	Biolegend (San Diego, USA)
CD14	M5E2	Alexa Fluor700	557923	BD (San José, CA, USA)
CD15	MMA	V450	642917	BD (San José, CA, USA)
CD19	89B (B4)	APC AlexaFluor700	A78837	BC (Marseille, France)
CD33	D3HL60.251	PE-Cy5.5	A70198	Immunotech (Marseille, France)
CD34	581	ECD	IM2709U	BC (Marseille, France)
CD34	8G12	APC	340441	BD (San José, CA, USA)
CD38	HIT2	perCP-cy5.5	551400	BD (San José, CA, USA)
CD45	HI30	Pacific Orange	MHCD4530	Invitrogen (Camarillo, USA)
CD56	N901	PE-CY7	A21692	BC (Marseille, France)
CD64	22	FITC	IM1604U	BC (Marseille, France)
CD117	104D2	PE	340529	BD (San José, CA, USA)
CD117	104D2	PE-CY7	339195	BD (San José, CA, USA)
HLA-DR	L243	APC-Cy7	307618	Biolegend (San Diego, USA)
cCD3	UCHT1	PB	558117	BD (San José, CA, USA)
TDT	HT-6	FITC	MHTDT01-5	Life technologies (Camarillo, USA)
CD79a	HM47	PE	IM2221	BC,(Miami, USA)
MPO	5B8	PE	341642	BD (San José, CA, USA)
Fix-and-Perm kit	-	-	GAS-002A-1<br>GAS-002B-1	Invitrogen (Camarillo, USA)

Panels for diagnosis of AML:
Tube1 CD64-FITC / CD7-PE / CD34-ECD / CD33-PE-CY5.5 / CD117-PE-CY7 / CD13-APC / CD14-APC-Alexa Fluor700 / HLA-DR-APC-CY7 / CD15-V450 / CD45-PO
Tube2 CD11B-FITC / CD117-PE / CD38-percp-cy5.5 / CD56-PE-CY7 / CD34-APC / CD19-APC-Alexa Fluor700/ CD4-APC-eFluor780 / CD2-PB / CD45-PO
Tube3 TdT-FITC / cMPO-PE / CD79a-PE-CY5 / CD34-ECD / CD117-PE-CY7 / cCD3-PB / CD45-PO

Notes: BD:Becton Dickinson & Company; BC: Beckman-Coulter

Supplementary Table 4: Primer sequences for the reference gene ABL and PML-RARA

	Forward 5′–3′	Probe 5′–3′	Reverse 5′–3′
PML-RARA-L	TCTTCCTGCCCAACAGCAA		
PML-RARA-S	ACCTGGATGGACCGCCTAG	AGTGCCCAGCCCTCCCTCGC	GCTTGTAGATGCGGGGTAGAG
PML-RARA-V	CCGATGGCTTCGACGAGTT		
ABL	TGGAGATAACACTCTAAGC<br>ATAACTAAAGGT	Fam-CCATTTTTGGTTTGGGC<br>TTCACACCATT-Tamra	GATGTAGTTGCTTGGGACCCA
